# Multidomain changes in clinical outcomes after 12 weeks of resistance-based exercise training in healthy older adults: a four‑domain framework for identifying non‑responders

**DOI:** 10.1007/s41999-026-01614-8

**Published:** 2026-09-22

**Authors:** Špela Matko, Barbara Strasser, Bibiane Steinecker-Frohnwieser, Tanja Stamm, Helmut Kern, Stefan Löfler, Stefanie Hochenwarter, Ferdinand Prüfer, Alexandra Unger, Veronika Tirpáková, Jan Cvecka, Nejc Šarabon, Vincent Grote

**Affiliations:** 1 Ludwig Boltzmann Institute for Rehabilitation Research, Reizenpfenninggasse 1, 1140 Vienna, Austria; 2 Institute for Outcomes Research, Medical University of Vienna, Vienna, Austria; 3 Faculty of Medicine, Sigmund Freud University Vienna, Vienna, Austria; 4 Ludwig Boltzmann Institute for Arthritis and Rehabilitation, Vienna, Austria; 5 Department of Orthopaedics and Trauma, Medical University of Graz, Graz, Austria; 6 Physiko and Rheumatherapie, Institute for Physical Medicine, St. Pölten, Austria; 7 University of Teacher Education, Klagenfurt, Austria; 8 Institute of Sports Medicine, Faculty of Medicine, Slovak Medical University, Bratislava, Slovakia; 9 Comenius University Bratislava, Faculty of Physical Education and Sport, Bratislava, Slovakia; 10 Masaryk University, Faculty of Sports Studies, Incubator of Kinanthropology Research, Brno, Czech Republic; 11 No Borders, CA (Civic Association), Holíč, Slovakia; 12 Department of Kinesiology, Faculty of Health Sciences, University of Primorska, Izola, Slovenia

**Keywords:** Healthy ageing, Resistance training, Rehabilitation outcomes, Multidomain assessment

## Abstract

**Purpose:**

To characterise multidomain changes observed over 12 weeks among healthy older adults participating in a supervised resistance-based exercise programme.

**Background:**

Resistance-based exercise improves physical performance in older adults, yet clinical evaluation often relies on isolated outcome measures and overlooks multidimensional changes. We investigated multidomain changes following a 12‑week supervised exercise training programme in healthy older adults using a reduced, clinically interpretable four‑domain framework and a Multidomain Quality Outcome Index (MQO_idx_) to characterise overall responsiveness.

**Methods:**

A total of 126 healthy older adults (age: 67.7  ±  5.3 years; 62.7% women) completed a 12-week training programme. A comprehensive battery of assessments was categorised in four clinically interpretable domains: ‘body composition’, ‘muscle capacity’, ‘functional performance’, and ‘pain & HRQoL’. Changes were expressed as *z*‑standardised factor scores and standardised mean differences (SMD). Multidomain responders were classified based on their MQO_idx_ change (≥  + .33 SMD).

**Results:**

Values in the ‘functional performance’ domain improved substantially (SMD  =  .67 ± .57), leading to overall improvement (MQO_idx_ SMD  =  .30 ± .56). Values in the ‘muscle capacity’ domain remained stable, while those in ‘body composition’ showed a slight negative shift. Changes in the ‘Pain & HRQoL’ domain were heterogeneous. Forty-six percent of participants met responder criteria. Non‑responders were characterised by worsening of values in the ‘pain & HRQoL’ domain and small deteriorations in the ‘muscle capacity’ and ‘body composition’ domains, while still exhibiting measurable improvements in ‘functional performance’. Baseline characteristics showed the strongest associations with ‘muscle capacity’ and ‘functional performance’, whereas associations with change were limited and primarily involved ‘pain & HRQoL’ and the composite index (MQO_idx_).

**Conclusions:**

Early-phase change in these four domains appears asynchronous. The use of a multidomain evaluation framework reveals this heterogeneity and may facilitate the early identification of diverging response trajectories. Baseline characteristics showed stronger associations with baseline domain status than with short-term changes. PROMs showed limited mean short-term change but contributed to identifying divergent outcome trajectories. As this was a single-arm study without a control group, the observed changes cannot be attributed to the intervention alone. Further studies are needed to assess external validity and longer-term stability.

Trial registration number: NCT05053282 (22.09.2021)

**Supplementary Information:**

The online version contains supplementary material available at https://doi.org/10.1007/s41999-026-01614-8.

## Key summary points

**Aim:**

We investigated multidomain changes observed over 12 weeks among healthy older adults participating in a resistance-based exercise programme.

**Findings:**

‘Functional performance’ improved consistently, whereas ‘muscle capacity’ and ‘body composition’ showed minimal changes, and ‘pain & HRQoL’ displayed heterogeneous trajectories. Multidomain outcome index revealed substantial interindividual variability, with almost half of participants classified as responders. Non‑responders were characterised by worsening in ‘pain & HRQoL’ and small deteriorations in ‘body composition’ and ‘muscle capacity’, while still demonstrating improved ‘functional performance’.

**Message:**

Multidomain outcome monitoring may support more comprehensive clinical evaluation of short-term outcome changes in healthy older adults and enable earlier identification of non‑response trajectories.

**Supplementary Information:**

The online version contains supplementary material available at https://doi.org/10.1007/s41999-026-01614-8.

## Introduction

Over the past decade, the global population aged 60 and older has risen sharply and is projected to reach approximately 2.1 billion by 2050 [1]. Although the higher life expectancy indicates the significant advances made in public health, these advances are not accompanied by proportional increases in Healthy Life Years (HLY) or Healthy Life Expectancy (HALE). These measures reflect years lived in good health rather than years survived [2–4]. Consequently, this older population faces increasing functional limitations and chronic conditions due primarily to noncommunicable diseases (43.8 million global deaths in 2021) that widen the lifespan and health span gap [5, 6].

A common age-related complication is the accelerated loss of muscle mass, muscle strength, and function known as sarcopenia [7]. Sarcopenia affects an estimated 10–26% of adults over the age of 60 [8] and is associated with a variety of negative health consequences, including increased mortality [9], limited functional independence [10], and reduced quality of life [11]. This complication is common among community-dwelling older adults, and especially among those who do not respond to physical activity recommendations or are recovering from injury or illness [12]. In parallel, modern healthcare providers are more strongly focusing on highly prevalent chronic conditions and recommending physical activity as a key therapeutic strategy, since an active lifestyle reduces the risk of chronic diseases by up to 50% [13]. Taken together, these trends underscore the growing significance of employing preventive, community-based strategies that integrate targeted physical activity with early and standardised monitoring of musculoskeletal health and functional capacity.

Resistance-based exercise training is considered a key non-pharmacological intervention for the prevention and treatment of age-related muscle loss and has been shown to improve muscle mass, muscle strength, neuromuscular function, and functional performance in older adults [14]. The strongest effects have been observed for parameters of physical performance, including gait speed, chair rise ability, and the Timed Up and Go (TUG) test [15]. Meta-analyses [16] show that progressive or multimodal strength training programmes can promote significant and robust improvements in muscle strength, mobility, and functional performance. Notably, these benefits are reflected in physiological parameters, functional performance, and health-related quality of life (HRQoL) [17]. However, another steadily accumulating body of evidence suggests that different outcome domains respond at different temporal rates, with early functional improvements frequently preceding measurable structural changes (e.g. neural-dominant early-phase responses) [18, 19]. This evidence supports the need for patient-centred approaches that allow the early detection of clinically meaningful changes and individual adjustments of interventions [20].

Despite the importance of standardised outcome assessments in research and clinical practice, current evaluation practices are limited by analytical and conceptual constraints. Frequent before-and-after comparisons of individual variables neglect the multidimensionality and heterogeneity of adaptive processes in older age, thereby limiting efforts both to interpret results and to stratify and personalise interventions [21]. These limitations are closely related to the insufficient consideration of baseline levels and interdependence between measurement variables. Baseline values have been shown to influence the direction and magnitude of longitudinal change, enable risk-based stratification, and define physiologically achievable adaptation ranges [22–24]. Studies in rehabilitation settings, for example, demonstrate that individuals with poorer functional or disease-specific baseline profiles frequently demonstrate larger absolute improvements after taking part in structured interventions [25]. Beyond this baseline dependency, additional methodological challenges arise due to inter-variable correlations and statistical artefacts, which can bias estimations of intervention effects if not explicitly addressed [26, 27].

Routine clinical assessments allow the identification of many partially redundant indicators in the physiology, functional performance, and HRQoL domains. While clinician-reported outcome measures (CROMs) reflect observable functions, patient-reported outcome measures (PROMs) reflect subjective experiences, which often correlate weakly with objective parameters and follow distinct trajectories [28, 29]. This divergence has also been observed in musculoskeletal rehabilitation settings, where integrated performance measures combining PROMs and CROMs have been proposed to improve the interpretability of multidimensional intervention effects [30]. Consequently, an isolated interpretation of individual endpoints may inadequately reflect multidimensional adjustments and result in less informed clinical decision-making [31, 32]. This is particularly evident in the context of healthy ageing, where muscle mass alone is increasingly recognised as an insufficient marker for functional impairment and treatment response [33].

Accordingly, current consensus definitions emphasise functional endpoints and call for an integrative consideration of objective, clinical, and patient-reported parameters [34]. The aggregation of such measures in cross-domain indices could enable a structured assessment of multidimensional intervention effects and the identification of early, clinically relevant changes [35]. The objective of this study was to characterise the multidomain changes observed over 12 weeks among healthy older adults participating in a supervised resistance-based exercise programme using a reduced four-domain framework and a composite Multidomain Quality Outcome Index (MQO_idx_). An additional aim was to explore multidomain response profiles using an a priori candidate responder threshold.

## Methods

### Study design and participants

We conducted a 12-week, longitudinal single-group intervention within the Center of Active Ageing Project (CAA) [36], performing standardised assessments at entry (T0) and after 12 weeks (T1).

Consecutive volunteers were recruited from 1 Nov. 2020 to 30 Sept. 2022 by advertising on the project website and in print media and flyers distributed in St. Pölten. The study included community-dwelling adults 60–80 years of age who were able to walk (with/without aids) and to read/speak German. Exclusion criteria included training-relevant musculoskeletal impairments; load-dependent pain ≥ 5 as measured with the visual analogue scale (VAS); body mass index (BMI) > 40 kg/m^2^; uncontrolled hypertension or diabetes; evidence of an acute systemic infection, acute cardiopulmonary disease in the prior 3 months; recent lower-limb deep vein thrombosis (<  3 months); vestibular disorders with dizziness/balance deficits; active malignancy under treatment; myopathy; neurological gait disorders (e.g. peripheral neuropathy, Parkinson’s disease); dementia/reduced cognition.

A total of 165 participants enrolled and took part in the training programme. Fourteen subjects were excluded because they lacked baseline diagnostic assessments (T0), resulting in a sample of 151 participants with complete T0 data. Of these, four participants discontinued the programme due to COVID-19 illness and three withdrew for personal reasons. A further 21 were excluded from the final analysis due to incomplete discharge (T1) diagnostic assessments. The final analytic sample comprised 126 (*N*) participants (Supplementary Figure S1).

### Intervention

Participants completed two supervised 60-min sessions/week for 12 weeks: one full-body resistance-training session and one neuromotor session. Resistance training followed a periodised progression referenced to estimated 1 repetition maximum (RM) [37]: weeks 1–3: 2 × 20 reps (75% 1RM), weeks 4–6: 2 × 12–15 (80% 1RM), weeks 7–12: 3 × 8–12 (85% 1RM). The training targeted the major upper/lower-limb and trunk muscle groups and featured individualised load progression. Neuromotor sessions included structured tasks for coordination, mobility/flexibility, and static/dynamic balance that became progressively more difficult. Sessions were delivered and supervised by a multidisciplinary team of qualified exercise professionals (e.g. physiotherapists and sport scientists). Together with the dietitians, this team provided a brief information session at the programme start and end (healthy lifestyle, safe exercise, nutrition, ageing-related risks, prevention).

### Outcome measures and assessment battery

The standardised admission (T0) and post-intervention (T1) assessment testing protocols adhered to the CAA Test protocol and covered the following: anthropometry and body composition; resting hemodynamic; functional performance of upper/lower extremities; walking capacity/speed and mobility; static and dynamic balance; joint and spinal mobility/flexibility; muscle strength of upper and lower limbs; generic health status/health-related quality of life (HRQoL) and symptom burden; nutritional risk screening; physical activity level; and medical history/medications, falls, and lifestyle factors. Assessments were performed by trained staff with calibrated equipment, and assessment results were recorded in the project database.

We assessed a broad battery of PROMs and CROMs, including body composition parameters (clinical/physiological). By applying a data-driven dimensionality reduction procedure (see 'Dimensionality reduction and composite outcome construction'), a total of 14 key outcome indicators were selected for inclusion in all subsequent analyses. The 14 indicators retained were BMI, ECM/BCM ratio, and body fat (‘body composition’); FFMI, BCM, isometric knee extensor torque, and handgrip strength (‘muscle capacity’); 30-s Chair Stand, TUG, and normal- and fast-paced 10-m walking speed (‘functional performance’); and EQ-5D pain, EQ-5D index, and SF-12 mental component summary (‘pain & HRQoL’). Operational details for the complete battery are found in Supplementary Table S1.

### Statistical analysis

All statistical analyses were conducted using IBM SPSS Statistics (Version 31). Prior to the analysis, the assumptions for parametric procedures were assessed by visually inspecting the histograms and Q–Q plots and by applying Levene’s test. These assumptions include normality and homogeneity of variances. No significant deviations from these assumptions were found. To standardise the measurements taken at different times, *z*-scores were calculated for each variable. These *z*-scores were based on the mean and standard deviation (SD) of the pooled data from T0 and T1. The score directions were harmonised to ensure that higher values consistently indicated improvement, regardless of the original metric orientation. In addition to these descriptive methods, parametric procedures were applied, including multifactorial repeated-measures analyses of variance (ANOVA); between-subject effects: sex, age, BMI; and within-subject effect: time (T0, T1), linear regressions, and Pearson correlations. Instead of relying solely on *p*-values, we primarily reported effect sizes [partial η^2^]. Partial η^2^ was reported given the sample size, where even small numerical differences could achieve statistical significance without clinical relevance. Effect sizes [38] were interpreted based on conventional thresholds: η^2^ = 0.01–0.06 (small), 0.06–0.14 (medium), > 0.14 (large). Dimensionality reduction decreased the number of outcomes and redundant tests but did not eliminate multiplicity. As the remaining analyses were exploratory, no formal adjustment for multiple comparisons was applied, and p-values were interpreted descriptively.

### Dimensionality reduction and composite outcome construction

**Fig. 1:**
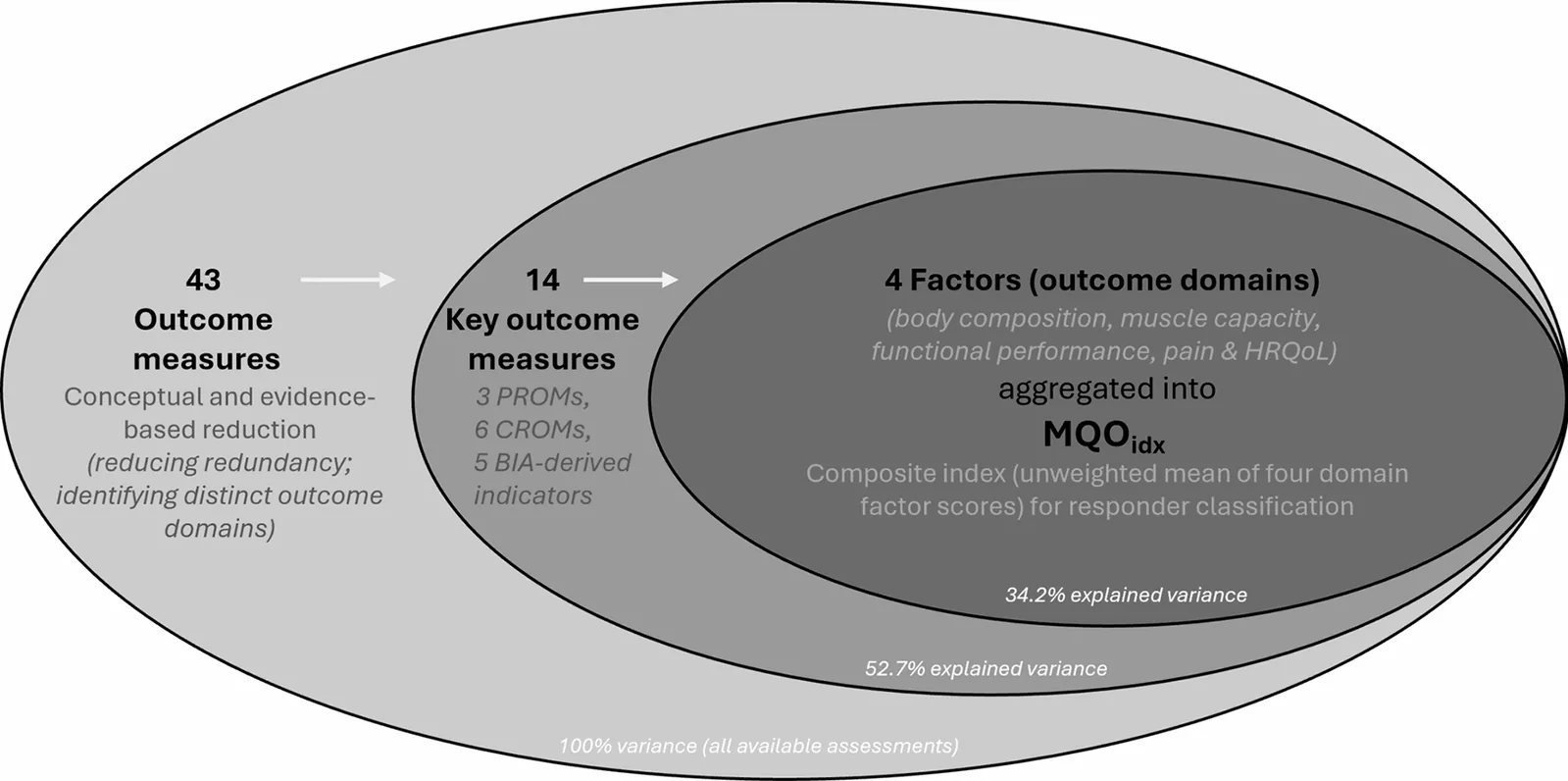
Multistep dimensionality reduction procedure and construction of the Multidomain Quality Outcome Index (MQO_idx_). Starting from 43 patient-reported, clinician-reported, and objective outcome measures, a reduced set of 14 key outcome indicators was identified using combined statistical and conceptual criteria. These indicators were subsequently aggregated into four latent outcome factor domains (‘body composition’, ‘muscle capacity’, ‘functional performance’, and ‘pain & HRQoL’) derived from principal component analysis. The four-domain factor scores were then combined into the Multidomain Quality Outcome Index (MQO_idx_), allowing the classification of the multidimensional rehabilitation responses. Percentages indicate the proportion of explained variance retained at each reduction step

To reduce redundancy among the correlated outcome measures and derive latent outcome domains, a multistep dimensionality reduction procedure based on principal component analysis (PCA [39]) was applied (Fig. 1) Starting from 43 outcome measures, a subset of 14 key outcome indicators was identified by applying PCA-based statistical criteria and considering conceptual aspects (see Supplementary Methods and Table S4). Although dimensionality reduction implies a loss of partial information, this approach was chosen to reduce measurement complexity and support the exploratory description of multidimensional response profiles. Potential selection bias was assessed by comparing available baseline characteristics between participants included in the final analysis (*n* = 126) and those not included (*n* = 39; see flowchart in Supplementary Figure S1), using independent-samples t-tests for continuous variables and Pearson’s Chi-square test for sex (Supplementary Table S7).

**Fig. 2:**
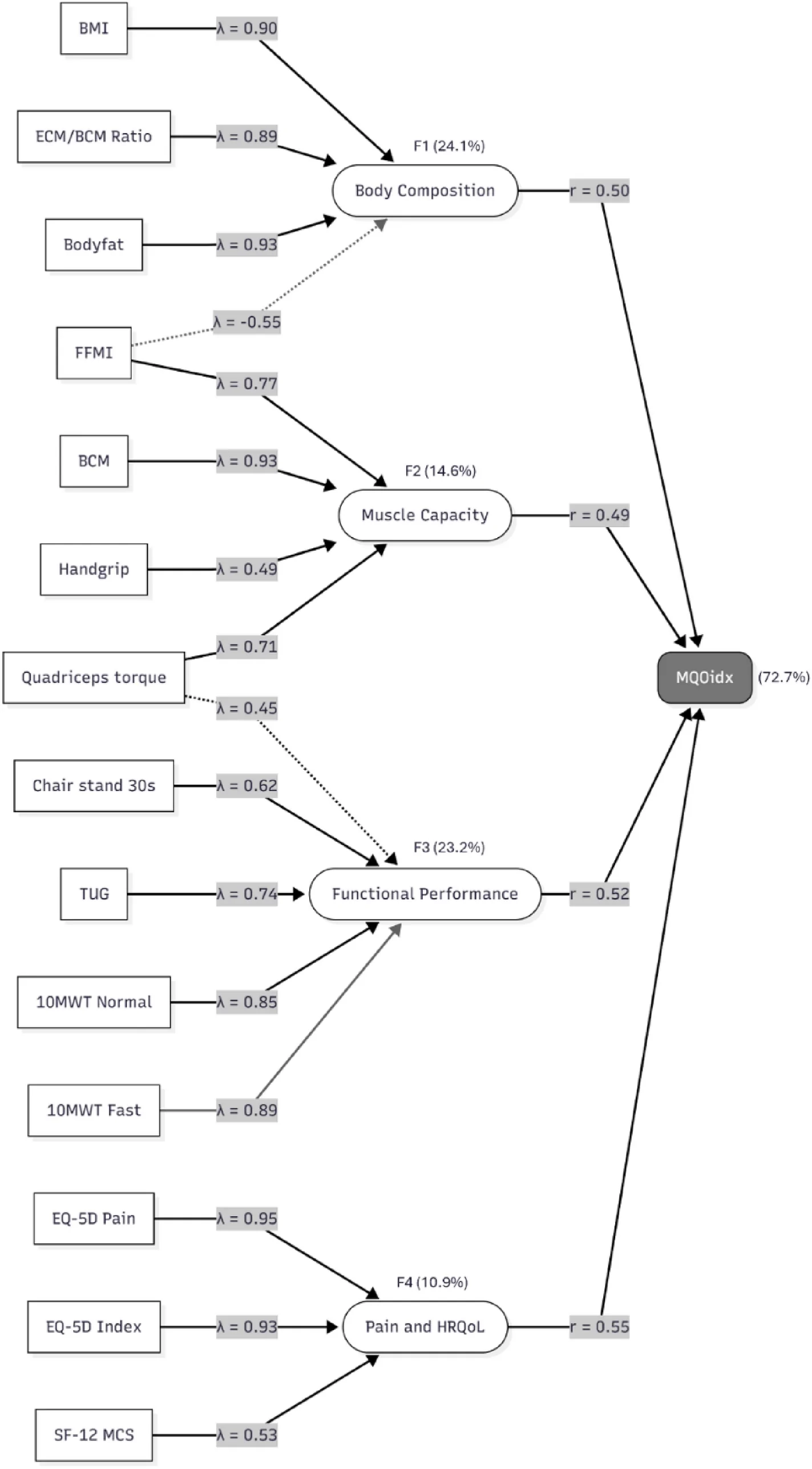
The conceptual structure of the four extracted domains derived from principal component analysis (PCA) with a varimax rotation. Four latent factor domains (F1-4) are shown as derived from principal component analysis (PCA) with a varimax rotation: ‘body composition’, ‘muscle capacity’, ‘functional performance’, and ‘pain & HRQoL’. Arrows represent standardised loadings (*λ*), which are used to represent the Pearson correlation coefficients between each indicator and the latent factor, and dotted lines indicate secondary loadings. The domains were extracted as orthogonal (statistically uncorrelated; all *p* > 0.05) components, but each showed a positive correlation with the overall MQO_idx_. The four-domain solution obtained with the factor analytic model shows a robust and coherent latent structure; the four factors jointly explain 72.7% of the variance (‘body composition’ 24.1%, ‘functional performance’ 23.2%, ‘muscle capacity’ 14.6%, ‘pain & HRQoL’ 10.9%) for the retained indicators

The retained indicators were subsequently aggregated into four latent outcome factor domains representing complementary dimensions of rehabilitation outcome: F1 ‘body composition’, F2 ‘muscle capacity’, F3 ‘functional performance’, and F4 ‘pain & HRQoL’ (Fig. 2). Standardised loadings (λ) representing the Pearson correlation coefficients between each indicator and the latent factor were used to interpret factor contributions. To obtain an overall outcome metric, the MQO_idx_ was calculated at each time point as the unweighted mean of the four-domain factor scores. Individual change was quantified as Δ = T1–T0 using *z*-standardised values, with higher values indicating improvement.

### Responder classification

In the context of descriptive reporting, the observed changes in each domain and the MQO_idx_ were then categorised according to the effect estimations (Cohen’s *d*/SMD) for the following criteria: ‘improved’ (SMD, Δ*z* > + 0.20), ‘stable’ (− 0.20 ≤ Δ*z* ≤ + 0.20), or ‘worsened’ (Δ*z* < − 0.20). The ± 0.20 thresholds were selected to retain sensitivity to small effects and highlight the between-domain differences in early-phase change. To enable the identification and differentiation of participants displaying a candidate criterion for multidimensional improvement, a binary responder classification was additionally applied. Participants were categorised as responders if MQO_idx_ ≥ + 0.33 SMD, and otherwise as non-responders. The one-third SD threshold has been reported as both perceptible and clinically meaningful in multidimensional rehabilitation outcomes [40]. It was selected a priori as a conservative, descriptive candidate threshold, and its application within the same derivation sample should not be interpreted as a validated clinical cut-off.

### Associations with baseline status and change—critical success factors (CSFs)

To explore factors associated with the baseline domain status and change in the 12‑week period, multiple linear regression models were applied. Separate models were calculated for baseline scores (T0) and change scores (Δ = T1–T0) for each domain (F1–F4) and the MQO_idx_. Candidate baseline variables were selected a priori based on clinical relevance and from the literature and included sex, age, BMI, the Global Physical Activity Questionnaire (GPAQ), nutritional status (MNA, Nutrition Index), systolic (SBP) and diastolic blood pressure (DBP), resting heart rate, and phase angle (PhA). SBP, DBP, and heart rate were included as general cardiovascular-health markers potentially associated with baseline exercise capacity, not as hypothesised direct causes of muscle function. To avoid redundancy and multicollinearity, the BMI, Nutrition Index, and PhA were excluded from the model’s ‘body composition’, ‘muscle capacity’, and MQO_idx_, as these constructs already included related physiological components. Baseline domain values were additionally entered as covariates in the change models to account for baseline dependency. Model fit was expressed using adjusted *R*^2^, and associations with *p* < 0.10 were reported as exploratory signals. These models were intended to describe associations rather than identify causal determinants or develop a prediction model.

## Results

### Participant characteristics

The analytic cohort comprised 126 healthy older adults (62.7% women) with a mean age of 67.7 ± 5.3 years and a mean BMI of 28.1 ± 4.5 kg/m^2^. Participants did not display acute or severe chronic conditions, representing a generally healthy and well-functioning population. Their nutritional status was deemed adequate according to the MNA, with 98.4% of participants scoring within the normal range ( ≥  24), and 1.6% classified as at risk of malnutrition; none of the participants were malnourished. The participants exhibited a mean PhA of 5.6 ± 0.64° and a mean fat-free mass index (FFMI) of 18.1 ± 2.3 kg/m^2^, indicating preserved ‘body composition’.

Based on sex-specific PhA cut-offs proposed by Akamatsu et al. [41], 4.8% (5 men, 1 woman) of participants were classified as “at risk” for sarcopenia. Men had significantly higher FFMI (20.1 ± 1.8 vs. 16.9 ± 1.7 kg/m^2^; *p* < 0.001) and PhA (5.85 ± 0.64 vs. 5.45 ± 0.59; *p* < 0.001) than women. No significant sex differences were observed according to age (*p* = 0.55), BMI (*p* = 0.28), and MNA (*p* = 0.73).

Supplementary Table S2 reports the baseline (T0) and post‑intervention (T1) values for the 14 key outcome indicators forming the four domains. Supplementary Table S3 provides descriptive values for the candidate baseline characteristics included in the exploratory association models, with T0 and T1 values reported where applicable: sex, age, BMI, GPAQ, Nutrition Index, MNA, SBP, DBP, resting heart rate, and PhA.

### Factor structure of domains

The factor analysis supported a four-domain structure comprising ‘body composition’, ‘muscle capacity, ‘functional performance’, and ‘pain & HRQoL’ that was consistent with the predefined dimensional reduction framework (Fig. 1) The rotated solution explained 72.7% of the variance within the retained indicator set. The regression coefficients derived from this solution were subsequently utilised to compute standardised factor scores. Detailed numerical results are reported in Supplementary Table S4, and the indicator–domain relationships are shown in Fig. 2.

The retained indicators demonstrated robust primary loadings (λ) based on their respective latent domains, supporting the conceptual grouping of variables. Minor secondary loadings were observed for FFMI and quadriceps torque, reflecting their overlapping relevance for both body composition and muscular performance. Orthogonal rotation was employed to ensure that the extracted domains were uncorrelated by design. In contrast, each domain exhibited moderate positive correlations with the MQO_idx_, suggesting that the four domains represent distinct yet complementary dimensions contributing to multidimensional rehabilitation outcomes.

### Primary outcomes: baseline scores and changes over time

Table 1 provides a concise overview of the baseline scores, mean changes, and time effects for all domains (F1–F4) and the MQO_idx_. A significant time effect was observed for the ‘body composition’ (*p* = 0.039, η^2^_p_ = 0.034) and ‘functional performance’ (*p* < 0.001, η^2^_p_ = 0.583) domains, as well as for the MQO_idx_ (*p* < 0.001, η^2^_p_ = 0.223). The improvements observed were reflected by positive mean differences (Δ = T1–T0), with the most pronounced gains evident in the ‘functional performance’ domain (SMD = 0.67 ± 0.57) and MQO_idx_, (SMD = 0.30 ± 0.56). No significant changes were identified in the ‘muscle capacity’ or ‘pain & HRQoL’ domains, demonstrating their relative stability in the rehabilitation period.

**Table 1:** Baseline scores, mean change, 95% confidence intervals (CI) and time effects for the domains and MQO_idx_(*n* = 126)

Outcome	Baseline T0 (mean ± SD)	Mean difference Δ (SMD ± SD)	95% CI (Δ)	Within-factor time (η²_p_)
F1 Body composition	.04 ± .96	−07 ± .38	[−.138; -.004]	.034*
F2 Muscle capacity	.01 ± .93	−02 ± .46	[−.102; .060]	.002
F3 Functional performance	−34 ± .90	.67 ± .57	[.570; .771]	.583***
F4 Pain & HRQoL	−01 ± .99	.02 ± 1.01	[−.161; .194]	.000
MQO_idx_	−15 ± .97	.30 ± .56	[.200; .396]	.223***

All factor scores were *z*-standardised, with higher values reflecting better outcomes. Δ = T1–T0 (positive = improvement); *p* = significance level of the within-factor time effect (repeated-measures ANOVA); η²_p_ = partial eta-squared. SMD = standardised mean difference. CI = 95% confidence interval for Δ (mean change). Asterisks denote significance levels: * *p* < .05; ** *p* < .01; *** *p* < .001.

No significant multivariate interaction effects were identified for Time × Sex (η²_p_ = .039), Time × Age (η²_p_ = .024), Time × BMI (η²_p_ = .062), or time × physical activity level (η²_p_ = .057). Subsequent univariate analyses revealed that these interactions did not attain statistical significance for individual domains.

### Categorical change and overall benefit

An analysis complementary to the mean change analyses was provided in which individual response patterns were examined based on standardised factor score differences (Δ = T1–T0). Participants were classified as ‘worsened’, ‘stable’, or ‘improved’ using predefined thresholds. As demonstrated in Table 2, ‘functional performance’ demonstrated the highest level of responsiveness, with 80.2% of participants classified as improved. An overall improvement according to the composite MQO_idx_ was observed in 59.5%. In contrast, most participants exhibited stability in ‘body composition’ (71.4%) and ‘muscle capacity’ (59.5%), while a smaller proportion demonstrated measurable gains (11.9 and 15.1%, respectively). Variability in changes in ‘pain & HRQoL’ was observed, with a comparable proportion of participants classified as improved (34.1%) and worsened (33.3%).

**Table 2:** Proportions of participants classified based on SMD-based thresholds as worsened, stable, or improved in the domains and MQO_idx_ (*n* = 126)

Domain	Worsened *n* (%)	Stable *n* (%)	Improved *n* (%)	T0–T1 correlation, r (T0–Δ correlation, r)
F1 body composition	21 (16.7)	90 (71.4)	15 (11.9)	.93*** (.03)
F2 muscle capacity	32 (25.4)	75 (59.5)	19 (15.1)	.91*** (.09)
F3 functional performance	4 (3.2)	21 (16.7)	101 (80.2)	.82*** (−18*)
F4 pain & HRQoL	42 (33.3)	41 (32.5)	43 (34.1)	.50*** (−48***)
MQO_idx_(.20)	22 (17.5)	29 (23.0)	75 (59.5)	.84*** (−22*)
Responder classification (.33)	*68 (54.0)*		58 (46.0)	

Δ = T1–T0 (positive = improvement). Change categories were based on standardised factor scores (*z*-scores): Δ ≤ -.20 = worsened, –.20 < Δ < +.20 = stable, Δ ≥ +.20 = improved. Responders were defined as participants with ΔMQO_idx_ ≥ + 33 (non-responders: ΔMQO < + 33). Values are presented as absolute frequencies and percentages (*n*, %). Pearson correlation coefficients were calculated between domain scores at T0 and T1, r(T0,T1), and between baseline domain scores and change scores, r(T0,Δ), where Δ = T1−T0. In Table 2, values outside parentheses represent r(T0,T1), while values in parentheses represent r(T0,Δ).

The T0–T1 correlations (Table 2) were *r* = .93 for ‘body composition’, *r* = .91 for ‘muscle capacity’, *r* = .82 for ‘functional performance’, and *r* = .50 for ‘pain & HRQoL’. The latter domain also showed greater variability in change scores (cf. Table 1) and a significant negative correlation between T0 and change (*r* = −.48, *p* < .001). The composite MQOidx showed a T0–T1 correlation of *r* = .80).

### Responder vs. non-responder analysis

**Fig. 3:**
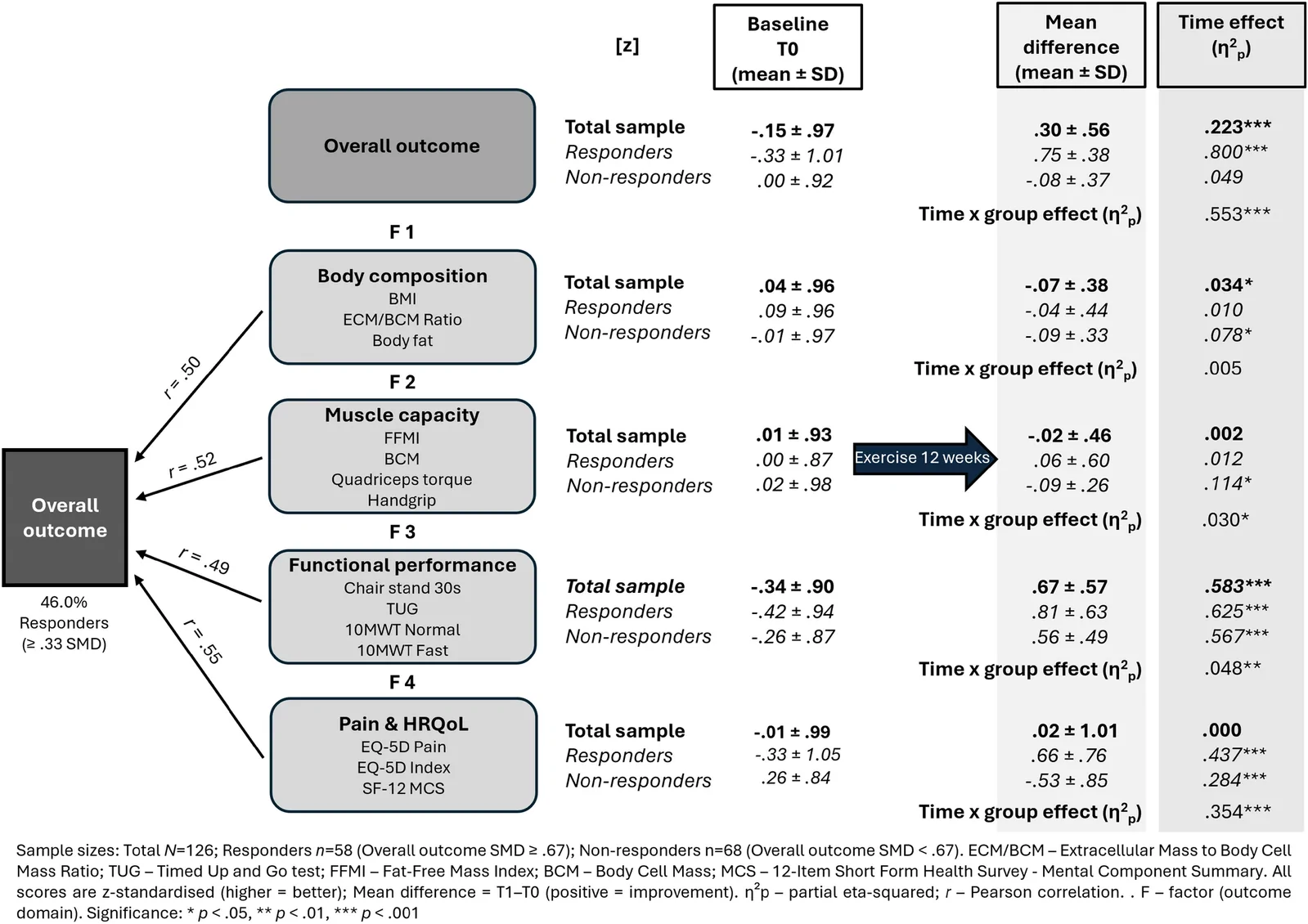
Baseline scores, mean differences, and time × group effects in responders (*n* = 58) and non-responders (*n* = 68). Responders were defined as participants with Δ*z* MQO_idx_ ≥ +0.33; non-responders had Δ*z* MQO_idx_ < +0.33. Factor scores are *z*-standardised (higher = better). Values are means ± SD. Δ = mean difference (T1–T0). η^2^_p_ = partial eta squared. Bold text—statistical significance. Asterisks indicate significance levels: * *p* < 0.05; ** *p* < 0.01; *** *p* < 0.001

To examine interindividual variability in observed change, participants were stratified into responders and non-responders based on changes in the MQO_idx_, applying a threshold of Δ*z* ≥ 0.33 to define clinically meaningful multidimensional improvement. Overall, 46.0% of participants were classified as responders. Descriptive statistics and effect size estimates are presented in Fig. 3.

At baseline, a significant between-group effect was observed for the ‘pain & HRQoL’ domain (*p* < 0.001, η^2^_p_ = 0.090), indicating worse baseline scores in responders compared with non-responders. No significant group differences were observed in ‘body composition’, ‘muscle capacity’, or ‘functional performance’ (*p* > 0.05).

**Fig. 4:**
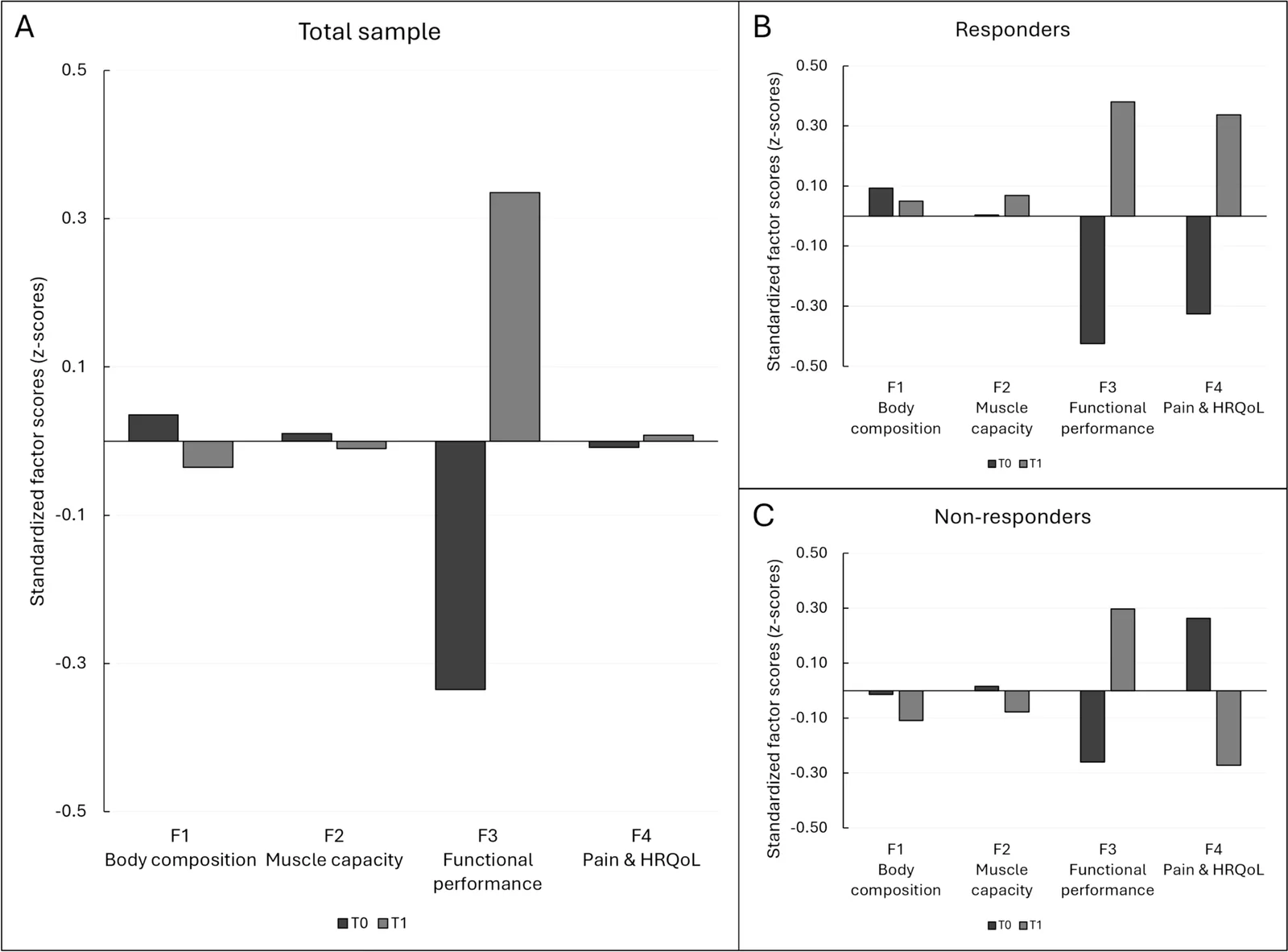
Standardised domain scores (*z*-scores) at admission (T0) and post-intervention (T1) in the total sample (*N* = 126), responders (*n* = 58) and non-responders (*n* = 68). The values represent z-standardised values for the change in domain (Δz = T1–T0), with higher values indicating better outcomes. Participants with ΔMQOidx ≥ +0.33 were defined as responders (non-responders: ΔMQO < +0.33). The results are presented for the total sample (**A**), the responders (**B**) and the non-responders (**C**)

The subsequent analysis revealed significant Time × Group interactions across multiple outcome domains, with responders exhibiting greater improvement than non-responders (Fig. 4). A pronounced interaction effect was observed for the MQO_idx_ (*p* < 0.001, η^2^_p_ = 0.553), reflecting divergent change trajectories characterised by substantial gains in responders and limited changes in non-responders. Furthermore, a substantial interaction effect was identified for ‘pain and HRQoL’ (*p* < 0.001, η^2^_p_ = 0.354) and ‘functional performance’ (*p* = 0.014, η^2^_p_ = 0.048), with ‘functional performance’ improving in both groups but showing larger gains in responders, whereas ‘pain & HRQoL’ improved in responders and worsened on average in non-responders. A smaller yet statistically significant interaction effect was identified for ‘muscle capacity’ (*p* = 0.050, η^2^_p_ = 0.030). Conversely, no substantial Time × Group interaction was evident in ‘body composition’ (*p* = 0.448, η^2^_p_ = 0.005).

### Associations with baseline status and longitudinal change

Multiple linear regression analyses were conducted to examine associations between candidate baseline characteristics and both baseline domain scores (T0) and changes over time (Δ T1–T0).

At baseline (T0), older age was associated with better ‘body composition’ (*β* = 0.201), whereas participants with higher systolic blood pressure tended to have poorer values (*β* = −0.223). For ‘muscle capacity’, males showed substantially higher scores than females (*β* = 0.891), while older participants showed lower scores (*β* = −0.145). For ‘functional performance’, older age (*β* = −0.275) and higher BMI (*β* = −0.230) were associated with poorer outcomes, whereas higher physical activity (GPAQ; *β* = 0.222) and better nutritional status (MNA; *β* = 0.184) were associated with better outcomes; diastolic blood pressure showed a positive trend (*β* = 0.182). No were significantly associated with ‘pain & HRQoL’. Males (*β* = 0.483) and participants with higher physical activity levels (GPAQ; *β* = 0.169) demonstrated better baseline MQO_idx_ values. Across domains, the explained variance was minimal for ‘body composition’ (adjusted *R*^2^ = 0.007) and ‘pain & HRQoL’ (adjusted *R*^2^ = −0.029), moderate for ‘functional performance’ (adjusted *R*^2^ = 0.229) and the MQO_idx_ (adjusted *R*^2^ = 0.292), and strong for ‘muscle capacity’ (adjusted *R*^2^ = 0.796) (see Supplementary Table S5).

For changes over time (Δ T1–T0), the explained variance of the domain-level models was largely absent, with adjusted *R*^2^ values ranging from − 0.024 to 0.192 across the ‘body composition’, ‘muscle capacity’, ‘functional performance’, and ‘pain & HRQoL’ domains. No baseline characteristics were associated with changes in ‘body composition’. Lower baseline GPAQ was associated with greater improvement in ‘functional performance’, while higher baseline GPAQ tended to be associated with greater improvement in ‘muscle capacity’. Participants with worse baseline ‘pain & HRQoL’ showed substantially larger improvements, whereas older individuals tended to show smaller gains. Lower baseline scores for the composite index were associated with greater overall improvement (*β* = − 0.323), where males showed larger gains than females (*β* = 0.219), and a higher resting heart rate showed a weak association with smaller improvements (*β* = −0.151). The explanatory value of this model was small but meaningful (adjusted *R*^2^ = 0.067). One important observation is that baseline ‘pain & HRQoL’ makes the strongest contribution to MQO_idx_ change, whereas no substantial baseline dependency was observed for changes in ‘body composition’, ‘muscle capacity’, or ‘functional performance’ (see Supplementary Table S6).

## Discussion

This study examined changes in healthy older adults following a 12-week resistance-based supervised exercise training programme in the domains of ‘body composition’, ‘muscle capacity’, ‘functional performance’ and ‘pain & HRQoL’, summarised as a composite index (MQO_idx_). By integrating objective performance indicators with subjective health measures, we aimed to describe multidimensional response heterogeneity relevant in geriatric medicine and identify non-responders, who were defined by the absence of meaningful improvement in the composite index.

Our study reveals domain‑specific and heterogeneous training‑associated changes in healthy older adults. The largest and most consistent improvement was seen in the ‘functional performance’ domain (Table 1), confirming the sensitivity of this tool for detecting short-term change in older adults. These findings are consistent with those of earlier research, indicating that mobility- and performance-based measures demonstrate a rapid response to resistance and neuromotor training stimuli [42–44]. Conversely, ‘muscle capacity’ and HRQoL showed on-average stability, while ‘body composition’ exhibited minor variability. The moderate increase in the composite MQO_idx_ suggests that functional gains were translated into broader multidomain benefits, also indicating that the early improvements were primarily in the neuromotor coordination rather than structural or subjective. These findings are clinically relevant for geriatric rehabilitation, where short-term interventions often prioritise improvements in mobility and functional independence.

Nevertheless, baseline performance provides relevant context regarding the functional status of the cohort. The participants demonstrated relatively elevated functional performance at T0: the mean 30-s Chair Stand result of 16.54 ± 4.40 repetitions was consistent with the published age- and sex-specific reference values for community-dwelling older adults [45], while the mean TUG time of 6.10 ± 0.90 s was faster than the pooled age-specific reference values reported for healthy adults aged 60–69 and 70–79 years [46]. Consequently, the recovery from marked baseline functional impairment is an implausible explanation for the observed changes. Furthermore, enhancements were evident across a range of complementary functional metrics, including the 30-s Chair Stand, the Timed Up and Go (TUG) test, and both usual and fast walking speed. This concordant pattern lends support to the interpretation of an overall improvement in functional performance during the observation period, as opposed to a change limited to a single test. However, the study does not establish intervention causality, and the influence of test familiarisation or other time-related factors cannot be excluded.

In addition, classifying individual changes as improved, stable, or worsened highlighted a marked heterogeneity across domains (see Table 2). While 80.2% of the participants demonstrated an improvement in ‘functional performance’, their responses in the ‘body composition’ and ‘muscle capacity’ domains were less pronounced, and substantial interindividual variability was observed in the ‘pain & HRQoL’ domain. Similar discrepancies between objective performance outcomes and PROMs have been reported in musculoskeletal rehabilitation and arthroplasty populations [28, 29, 47]. PROMs can be used to examine health dimensions other than physical capacity, including symptom perception, emotional wellbeing, and daily functioning. Changes detected using PROMs may therefore follow different temporal trajectories than those of clinician-reported or performance-based measures [31, 32]. This variability is consistent with the multifactorial nature of perceived health, which is influenced not only by physical adaptation but also by psychological, social, and contextual factors. Consequently, reliance on PROMs alone may provide an incomplete or potentially misleading picture of observed outcome profile. Instead, subjective outcomes should be interpreted with clinician-reported performance measures and objective physiological indicators to provide a more comprehensive and clinically meaningful evaluation of intervention effects in older adults.

In this study, the observed pattern of early functional improvements without parallel structural changes is consistent with evidence that neural adaptations dominate early phases of resistance training, whereas hypertrophic responses typically require longer intervention periods or higher training intensities [48, 49]. The low prevalence of sarcopenia risk in this cohort, as indicated by preserved PhA values, provides additional support for the interpretation that the baseline muscle health of our study participants was relatively high. In this context, resistance-based exercise has been shown to primarily enhance neuromuscular efficiency and coordination, thereby leading to functional gains even in the absence of substantial changes in muscle mass. These findings support the prioritisation of performance-based outcomes when evaluating short-term preventive interventions in healthy ageing populations [50].

The classification of responders was based on the composite MQO_idx_, which provided additional insights into interindividual variability. Approximately half (46%) of the participants fulfilled the responder criterion. The baseline differences were observed exclusively in the ‘pain & HRQoL’ domain, where responders reported poorer subjective health. In contrast, no significant differences between the groups were seen in the objective domains (‘body composition’, ‘muscle capacity’, ‘functional performance’). Our findings suggest that the responder status was predominantly influenced by the baseline symptom burden and the potential for improvement, rather than by overall functional or physiological impairments. One especially interesting result was that only the ‘pain & HRQoL’ domain differed at baseline between responders and non‑responders, whereas no significant group differences were seen for the objective domains (‘body composition’, ‘muscle capacity’, ‘functional performance’). However, ‘functional performance’ improved in both groups, indicating that this domain reflects a universal short-term change rather than serves as a discriminator of responder status. In contrast, non‑responders exhibited small yet consistent examples of deterioration across several domains, suggesting asynchronous multidomain adaptation rather than an absence of functional benefit. From a clinical standpoint, the robustness of functional gains is directly related to healthy ageing indicators such as HLY and HALE. In contrast, the structural and subjective domains appear to be more susceptible to variability in early-phase improvements. Consequently, multidomain monitoring (i.e. other than performance monitoring) may be required to identify individuals whose structural or subjective trajectories deteriorate although their functional improvement remains the same.

Baseline characteristics may operate as critical success factors (CSFs; [51]), shaping both the initial clinical profile and the capacity for change across domains. A strong baseline dependency was evident for ‘muscle capacity’, where demographic and anthropometric characteristics explained most interindividual variability, whereas ‘functional performance’ and ‘pain & HRQoL’ showed only weak associations with the same factors. The constraining influence of baseline levels on the magnitude and direction of observed change has been repeatedly documented in rehabilitation science and aligns with established descriptions of baseline-dependency and regression-to-the-mean phenomena [22, 24, 27]. In the analysis of change scores, only a small subset of baseline values showed exploratory associations: poorer baseline ‘pain & HRQoL’ and lower overall index were associated with greater improvements, while no meaningful success factors emerged for changes in ‘body composition’, ‘muscle capacity’, or ‘functional performance’. These findings suggest that baseline status is associated with the magnitude of observed change but has limited explanatory value for interindividual differences in responsiveness. In geriatric populations where baseline function has been maintained, the presence of ceiling effects can further restrict the observable improvement, thereby influencing the interpretation of intervention efficacy. In this context, static minimal clinically important difference (MCID) thresholds derived from clinical populations may not transfer well to preventive cohorts, where smaller but meaningful changes are expected. More flexible, population‑specific thresholds may therefore provide a more accurate evaluation of early‑phase training effects.

These findings should be interpreted considering several limitations. First, the single-group pre–post-design without a non-exercising control group restricts the study’s external validity. The observed improvements, particularly in ‘functional performance’ and overall index, cannot be categorically attributed to the intervention and may partly reflect spontaneous change, learning/familiarisation effects (especially in performance-based assessments like walking speed or chair rise tests), or regression to the mean. Second, the participants were healthy, community-dwelling older adults who were self-selected and had a preserved baseline status. This restricts the generalisability of the findings to frailer or multimorbid populations and may have contributed to ceiling effects, especially in terms of ‘pain & HRQoL’. Third, the final analyses included 126 of the 165 enrolled participants. Although the baseline comparison showed no consistent pattern of differences across most available characteristics, isolated differences in body fat, and residual selection bias cannot be excluded (Supplementary Table S7). In addition, recent hospitalisation or immobilisation during the preceding 3 months was not a formal exclusion criterion and was not systematically recorded; therefore, its potential influence on baseline functional status and observed changes cannot be excluded. Fourth, the 12-week duration and training dose were chosen for feasibility rather than to maximise morphological adaptation. ‘Body composition’ was also assessed using bioelectrical impedance rather than reference imaging. Therefore, the absence of consistent changes in ‘body composition’ or ‘muscle capacity’ should not be interpreted as evidence of definitive non-responsiveness of these domains. Fifth, although dimensionality reduction reduced redundant testing, no formal adjustment for multiple comparisons was applied, and false-positive findings therefore remain possible. Finally, the four-domain structure, the MQOidx and the responder threshold were derived and applied to the same sample using pooled T0 and T1 data. However, this in-sample model development may have resulted in optimistic estimates of model stability, which may have contributed to the observed separation between responders and non-responders, particularly for the MQOidx, on which the classification was based. Therefore, external validation and anchor-based calibration in independent cohorts are required before clinical application.

## Conclusion

By applying the reduced four-domain framework together with the composite MQO_idx_, we identified heterogeneous multidomain response profiles, including non-responders who nevertheless showed measurable functional gains. This underscores the potential clinical value of multidomain outcome assessment in healthy ageing, as it can reveal unfavourable early-phase trajectories that may remain undetected when interpretation relies on functional performance alone. Future research should externally validate the MQO_idx_ and candidate responder threshold in independent geriatric cohorts and determine whether early changes in pain and HRQoL predict longer‑term multidomain outcomes and could inform timely adjustments of exercise prescriptions. This also suggests that MCID and responder thresholds should be interpreted in relation to the outcome domain and assessment period, as some outcomes may be less responsive within 12 weeks.

## Supplementary Information

Below is the link to the electronic supplementary material.

Supplementary file1 (DOCX 213 KB)

## Data Availability

The research data supporting this publication are stored at our institutional digital data repository for published research accessible at https://creed.lbg.ac.at (accessed on 19 February 2026). The data sets analysed in this manuscript are not publicly available due to ethical and legal restrictions. (Data contain potentially identifying and sensitive patient information.) However, pseudonymised datasets have been created for the purpose of re-use and are also accessible at creed.lbg.ac.at. Requests for access to anonymised datasets should be directed to the corresponding author [VG].
