# Supplementary material for: Multidomain changes in clinical outcomes after 12 weeks of resistance-based exercise training in healthy older adults: a four‑domain framework for identifying non‑responders
Source: Eur Geriatr Med. 2026 Sep 22;17(5):2931–44. doi: 10.1007/s41999-026-01614-8 (PMC13754974; doi:10.1007/s41999-026-01614-8)
Supplement: Supplementary file 1 [file 41999_2026_1614_MOESM1_ESM.docx]

1. Appendix 1. Supplement

**Supplementary methods**

***Principal component analysis (PCA) and indicator selection***

The data structure was examined using principal component analysis (PCA) based on the correlation matrix with a varimax rotation to identify the underlying domains of the patient-reported, clinician-reported and objective measures. The Kaiser criterion (eigenvalues > 1) and the subsequent inspection of the scree plot were utilised to determine the number of components to be retained. The adequacy and suitability of the samples for PCA were confirmed using the Kaiser–Meyer–Olkin (KMO) measure, Bartlett's test of sphericity, and the determinant of the correlation matrix to rule out multicollineararity. Items with factor loadings below .50 were excluded from the model, as they did not contribute meaningfully to any factor. To guarantee a straightforward and readily interpretable factor structure, variables demonstrating substantial cross-loadings were also eliminated. Cross-loadings were defined as loadings ≥ .30 on more than one factor or as cases where the difference between the primary and secondary loadings was < .20, indicating ambiguous factor affiliation. Furthermore, variance loss was meticulously monitored during item reduction to ensure that the exclusion of variables did not substantially decrease the total explained variance or compromise the conceptual coherence of the final model. The final PCA model incorporated 14 standardised indicators and yielded a four-factor solution (Supplementary Table S4).

**Supplementary Figure S1:** Flowchart of participants’ inclusion and exclusion criteria

Overview of recruitment, baseline assessment (T0), intervention completion, post-intervention assessment (T1), and derivation of the final analytic sample.

**Supplementary Table S1.** Operational details of the full assessment

| **Measure type** | **Assessment** | **Unit** | **Device / Manufacturer** |
| --- | --- | --- | --- |
| PROM | *EuroQol 5 – Mobility* | 1–5 | Paper questionnaire |
|  | *EuroQol 5 – Self-care* | 1–5 |  |
|  | *EuroQol 5 – Usual activities* | 1–5 |  |
|  | **EuroQol 5 – Pain** | 1–5 |  |
|  | EuroQol 5 *–* Anxiety/depression | 1–5 |  |
|  | EuroQol 5 *–* Raw score | 5–25 |  |
|  | **EuroQol 5** *–* **Index** | 0–1 |  |
|  | *EuroQol 5 – Visual Analogue Scale (VAS)* | 0–100 |  |
|  | *12-item short form health survey — physical component summary (SF-12 PCS)* | 0-50 |  |
|  | **12-item short form health survey — mental component summary (SF-12 MCS**) | 0-50 |  |
|  | *Mini Nutritional Assessment (MNA)* | 0-30 |  |
|  | *Global Physical Activity Questionnaire — MET minutes (GPAQ MET)* | MET-min/week |  |
| CROM | ***Maximal isometric handgrip strength — mean (handgrip mean)*** | kg | Jamar Hydraulic Hand Dynamometer, model 5030J1 (Performance Health—JAMAR®, Warrenville, IL, USA) |
|  | **Isometric knee extensor torque — mean (quadriceps torque)** | kg | Isometric knee dynamometer (“Knee Dynamometer”; S2P d.o.o.—Science to Practice, Ljubljana, Slovenia) |
|  | Isometric knee extensor torque — mean (quadriceps torque/weight mean) | Nm/kg |  |
|  | **30-second Chair Stand test (30-s Chair Stand)** | repetitions | Standard chair; stopwatch |
|  | *30-second Arm Curl test (30-s Arm Curl)* | repetitions | Dumbbell (3 kg for men, 1 kg for women); stopwatch |
|  | *2-minute Step test (2-Min Step Test)* | number of steps | Stopwatch |
|  | *Chair Sit-and-Reach Flexibility Test (Sit-and-Reach)* | cm | Sit-and-reach box or measuring tape |
|  | *Back Scratch test* | cm | Measuring tape |
|  | *Single-leg Stance Test performed with eyes open — Mean* | s | Stopwatch |
|  | *Single-leg Stance Test performed with eyes closed — Mean* | s | Stopwatch |
|  | **Timed Up and Go test (TUG)** | s | Standard chair; 3-m walkway; stopwatch |
|  | 10-Meter Walk Test — normal pace (10MWT usual time) | s | Marked 10-m course; stopwatch |
|  | **10-Meter Walk Test — normal pace (10MWT usual speed)** | m/s |  |
|  | 10-Meter Walk Test — fast pace (10MWT fast time) | s |  |
|  | **10-Meter Walk Test — fast pace (10MWT fast speed)** | m/s |  |
| Clinical/ physiological | **Body Mass Index (BMI)** | kg/m² | Calibrated scale and stadiometer |
|  | Waist circumference | cm | Measuring tape |
|  | Hip circumference | cm |  |
|  | *Waist-to-hip ratio (WHR)* | ratio |  |
|  | Resting energy expenditure (REE) | kcal/day | BIA device (e.g. InBody 770) or indirect calorimeter |
|  | **Fat-Free Mass Index (FFMI)** | kg/m² |  |
|  | **Total body fat (kg body fat)** | kg |  |
|  | Total body fat percentage (Body fat %) | % |  |
|  | Extracellular mass (ECM) | kg |  |
|  | **Body cell mass (BCM)** | kg |  |
|  | **Extracellular-to-body-cell mass ratio (ECM/BCM)** | ratio |  |
|  | *Phase angle (PhA)* | degrees (°) |  |
|  | *Composite Nutrition Index (Nutrition Index)* | index |  |
|  | *Systolic blood pressure (SBP)* | mmHg | Automated sphygmomanometer |
|  | *Diastolic blood pressure (DPB)* | mmHg |  |
|  | *Resting heart rate (HR)* | bpm |  |

PROMs – patient-reported outcome measures; CROMs – clinician-reported performance tests.

Variables retained for the final factor analysis (*n* = 14) are highlighted in bold. Variables shown in italics were excluded due to insufficient communality (*h*² ≤ .50). The remaining variables were excluded during the selection process due to redundancy, high intercorrelations, or conceptual overlap with other measures. Handgrip strength represents an exception to the communality criterion and was retained although *h*² < .50 due to its strong clinical relevance and its frequent use and reporting in studies involving older adults.

**Supplementary Table S2.** Baseline (T0) and post‑intervention (T1) values of the 14 key outcome indicators

| **Domain** | **Outcome measure** | **T0  (Mean ± SD)** | **T1  (Mean ± SD)** | **Δ  (Mean ± SD)** |  |
| --- | --- | --- | --- | --- | --- |
| **F1  Body composition** | BMI [kg/m²] | 28.09 ± 4.52 | 28.14 ± 4.65 | .05 ± .75 |  |
|  | ECM/BCM ratio | 1.16 ± .24 | 1.17 ± .23 | .01 ± .15 |  |
|  | Body fat [%] | 34.86 ± 6.98 | 34.58 ± 7.13 | −.28 ± 2.21 |  |
| **F2  Muscle capacity** | FFMI [kg/m²] | 18.11 ± 2.35 | 18.22 ± 2.46 | .11 ± .63 |  |
|  | BCM [kg] | 23.50 ± 4.18 | 23.65 ± 4.32 | .15 ± 1.14 |  |
|  | Torque mean [Nm] | 130.12 ± 40.17 | 136.24 ± 41.74 | 6.13 ± 14.12 |  |
|  | Handgrip mean [kg] | 34.09 ± 9.62 | 34.97 ± 10.27 | .88 ± 3.43 |  |
| **F3  Functional performance** | FT Chair-30s [rep] | 16.54 ± 4.40 | 19.15 ± 4.90 | 2.61 ± 3.56 |  |
|  | TUG [s] | 6.10 ± .90 | 5.71 ± .95 | −.39 ± .59 |  |
|  | 10-m normal speed [m/s] | 1.26 ± .17 | 1.40 ± .18 | .13 ± .14 |  |
|  | 10-m fast speed [m/s] | 1.70 ± .23 | 1.83 ± .25 | .13 ± .17 |  |
| **F4  Pain & HRQoL** | EQ-5D Pain [1–5] | 1.65 ± .64 | 1.64 ± .68 | −.01 ± .70 |  |
|  | EQ-5D Index [0–1] | .94 ± .06 | .94 ± .06 | .00 ± .07 |  |
|  | SF-12 MCS | 79.62 ± 10.96 | 82.09 ± 9.89 | 2.47 ± 10.24 |  |

Values are presented as mean ± standard deviation (SD). Δ represents the change from baseline to post-intervention (T1–T0). Units are reported in square brackets. The 14 indicators reflect the reduced set of key outcome measures selected through the PCA-based dimensionality reduction procedure and used to construct the four domains (‘body composition’, ‘muscle capacity’, ‘functional performance’, ‘pain & HRQoL’) and the composite Multidomain Quality Outcome Index (MQO_idx_).

BMI – Body Mass Index; FFMI – Fat‑Free Mass Index; BCM – body cell mass; ECM/BCM – extracellular‑to‑body‑cell mass ratio; body fat % – body fat percentage; torque mean – isometric knee extensor torque; handgrip mean – isometric handgrip strength; FT Chair‑30s – 30‑Second Chair Stand Test; TUG – Timed Up and Go test; 10-m normal/fast speed – 10‑meter Walk Test speed at usual/fast pace; EQ‑5D Pain – EuroQol 5‑Dimensions Questionnaire pain dimension; EQ‑5D Index – EuroQol 5‑Dimensions Summary Index; SF‑12 MCS – 12‑item short form mental component summary.

**Supplementary Table S3.** Baseline (T0) and post-intervention (T1) values of candidate variables included in the exploratory association analyses

| **Outcome measure** | **T0 (Mean ± SD)** | **T1 (Mean ± SD)** | **Δ (Mean ± SD)** |
| --- | --- | --- | --- |
| BMI [kg/m²] | 28.09 ± 4.52 | 28.14 ± 4.65 | .05 ± .75 |
| GPAQ_MET [MET·min/week] | 5396.73 ± 3301.13 | 6854.57 ± 4322.01 | 1457.84 ± 4758.66 |
| Nutrition Index [%] | 98.06 ± 11.23 | 98.01 ± 13.68 | -.06 ± 12.82 |
| MNA [] | 27.41 ± 1.53 | 27.47 ± 1.67 | .06 ± 1.93 |
| SBP [mmHg] | 138.74 ± 18.45 | 134.29 ± 17.45 | -4.45 ± 13.92 |
| DBP [mmHg] | 81.06 ± 11.16 | 79.33 ± 10.70 | -1.72 ± 9.00 |
| HR [beat/min] | 70.25 ± 10.23 | 69.89 ± 9.34 | -.36 ± 8.23 |
| PhA [°] | 5.60 ± .64 | 5.64 ± .60 | .04 ± .44 |

Values are presented as mean ± standard deviation (SD). Δ represents the change from baseline to post‑intervention (T1−T0). BMI – body mass index; GPAQ_MET – Global Physical Activity Questionnaire (MET‑min/week); MNA – Mini Nutritional Assessment; SBP – systolic blood pressure; DBP – diastolic blood pressure; HR – resting heart rate; PhA – phase angle.

**Supplementary Table S4:** Factor structure of the four domains based on combined admission (T0) and post-intervention (T1) data (*N* = 252)

| Domain | Variable | Communality | Factor  loading (λ) | Explained  variance  (%) | Beta  coefficient | |
| --- | --- | --- | --- | --- | --- | --- |
| **F1  Body composition** | BMI | .877 | .902 | 24.1 | .307 | |
|  | ECM/BCM Ratio | .789 | .887 |  | .387 | |
|  | Body fat | .876 | .926 |  | .409 | |
| **F2  Muscle capacity** | FFMI | .898 | .768 | 14.6 | .221 | |
|  | BMC | .887 | .931 |  | .643 | |
|  | Handgrip | .281 | .490 |  | .195 | |
|  | Quadriceps torque | .770 | .711 |  | .106 | |
| **F3  Functional performance** | Chair stand 30s | .486 | .615 | 23.2 | .208 | |
|  | TUG | .653 | .741 |  | .257 | |
|  | 10MWT normal | .730 | .845 |  | .379 | |
|  | 10MWT fast | .816 | .887 |  | .360 | |
| **F4  Pain & HRQoL** | EQ-5D Pain | .902 | .947 | 10.9 | .457 | |
|  | EQ-5D Index | .873 | .933 |  | .455 | |
|  | SF-12 MCS | .351 | .528 |  | .232 | |
| **72.7% Total variance explained** | | | | | |  |

BCM – body cell mass; FFMI – Fat-Free Mass Index; BMI – Body Mass Index; ECM/BCM – extracellular-mass-to-body cell mass ratio; TUG – Timed Up and Go test; 10MWT normal/fast – 10-meter Walk Test at Normal/Fast Speed; EQ-5D Pain – EuroQol 5-Dimensions Questionnaire pain dimension; EQ-5D Index – EuroQol 5-Dimensions Questionnaire Summary Index; MCS – 12-item short form health survey — mental component summary.

Reported values are standardised factor scores. Communality = variance explained per variable; factor loading (λ) = standardised factor loading; corresponds to the Pearson correlation coefficient between the indicator and the latent factor; variance explained = percentage of variance accounted for by the factor; beta coefficient = regression weight used for factor score computation.

β is the standardised regression coefficient from stepwise multiple regression within each domain. It reflects the relative contribution of each indicator to the composite domain score. Regression coefficients (B) were utilised to calculate factor scores as weighted linear composites of *z*-standardised indicators.

Two indicators showed moderate cross-loadings: FFMI (primary loading on ‘muscle capacity’ λ = .77; secondary loading on ‘body composition’ λ = −.55) and quadriceps torque (primary loading on ‘muscle capacity’ λ = .71; secondary loading on ‘functional performance’ λ = .45). Both were retained based on their theoretical relevance and clinical interpretability (see Fig. 2). The final four-factor structure proved to be both coherent and stable.

Starting from 43 initially assessed outcome measures, a reduced set of 14 key indicators was selected, retaining 52.7% of the variance of the original 43-variable measurement space. This step reflects the efficiency of the variable selection procedure in preserving information while reducing measurement complexity.

The selected indicators were subsequently aggregated into four conceptually defined domains (‘body composition’, ‘muscle capacity’, ‘functional performance’, and ‘pain & HRQoL’). Representing these 14 indicators by four latent domains accounted for 34.2% of the variance within the original measurement space, reflecting the intended abstraction from individual indicators to higher-level constructs.

Within the factor analytic model, the four-domain solution showed a robust and coherent latent structure, with the four factors jointly explaining 72.7% of the variance (‘body composition’ – 24.1%, ‘functional performance’ – 23.2%, ‘muscle capacity’ – 14.6%, ‘pain & HRQoL’ – 10.9%) for the retained indicators.

**Supplementary Table S5:** Multiple linear regression analyses of associations with baseline domain scores and composite index (MQO_idx_) scores (T0)

| Domain T0 | Baseline variable | *B* (SE) | β | *p* | adj. *R*² |
| --- | --- | --- | --- | --- | --- |
| **F1 Body composition** | Sex | -.093 | -.047 | .612 | .007 |
|  | Age | .037 | .201 | **.037** |  |
|  | GPAQ | < .001 | .099 | .285 |  |
|  | MNA | -.050 | -.080 | .379 |  |
|  | SBP | -.012 | -.223 | **.062** |  |
|  | DBP | .011 | .133 | .267 |  |
|  | HR | .009 | .093 | .303 |  |
|  |  |  |  |  |  |
| **F2 Muscle capacity** | Sex | 1.701 | .891 | **<.001** | .796 |
|  | Age | -.026 | -.145 | **.001** |  |
|  | GPAQ | < .001 | -.031 | .459 |  |
|  | MNA | .002 | .004 | .932 |  |
|  | SBP | 0 | -.007 | .897 |  |
|  | DBP | .001 | .007 | .904 |  |
|  | HR | -.002 | -.025 | .541 |  |
|  |  |  |  |  |  |
| **F3 Functional  performance** | Sex | .222 | .119 | .173 | .229 |
|  | Age | -.047 | -.275 | **.003** |  |
|  | BMI | -.046 | -.23 | **.006** |  |
|  | GPAQ | < .001 | .222 | **.008** |  |
|  | Nutrition Index | .071 | .884 | .600 |  |
|  | MNA | .108 | .184 | **.025** |  |
|  | SBP | .001 | .031 | .775 |  |
|  | DBP | .015 | .182 | **.090** |  |
|  | HR | .007 | .077 | .345 |  |
|  | PhA | -1.278 | -.900 | .592 |  |
|  |  |  |  |  |  |
| **F4 Pain & HRQoL** | Sex | .121 | .060 | .554 | -.029 |
|  | Age | -.016 | -.086 | .407 |  |
|  | BMI | -.011 | -.049 | .602 |  |
|  | GPAQ | < .001 | .031 | .742 |  |
|  | Nutrition Index | -.127 | -1.442 | .459 |  |
|  | MNA | .022 | .034 | .716 |  |
|  | SBP | .010 | .186 | .134 |  |
|  | DBP | -.006 | -.068 | .579 |  |
|  | HR | .005 | .053 | .577 |  |
|  | PhA | 2.35 | 1.521 | .434 |  |
|  |  |  |  |  |  |
| **MQO_idx_** | Sex | .966 | .483 | **<.001** | .292 |
|  | Age | -.022 | -.117 | .149 |  |
|  | GPAQ | < .001 | .169 | **.032** |  |
|  | MNA | .035 | .055 | .476 |  |
|  | SBP | -.002 | -.033 | .741 |  |
|  | DBP | .012 | .144 | .157 |  |
|  | HR | .010 | .109 | .154 |  |

Values are reported as standardized regression coefficients (β), *p*-values, and adjusted *R*² for the final models. Sex was coded as 0 = female and 1 = male. Higher domain scores indicate better baseline status. BMI, Nutrition Index, and PhA were excluded from the regression models for ‘body composition’, ‘muscle capacity’, and MQO_idx_ to reduce redundancy and multicollinearity. Bold text - trend-level associations indicated by *p* < .10.

Negative adjusted R² values occurred only for the ‘pain & HRQoL’, indicating that the model did not explain baseline variability in this domain.

BMI – Body Mass Index; GPAQ – Global Physical Activity Questionnaire; Nutrition Index – Nutritional Index Score; MNA – Mini Nutritional Assessment; SBP – systolic blood pressure; DBP – diastolic blood pressure; HR – heart rate; PhA – phase angle.

**Supplementary Table S6:** Multiple linear regression analyses of associations with changes in domain scores and composite index (MQO_idx_) scores (Δ = T1-T0)

| Domain change | Baseline variable | *B* (SE) | β | *p* | adj. *R*² |
| --- | --- | --- | --- | --- | --- |
| **F1 Body composition** | Baseline F1 | -.011 | -.027 | .824 | -.024 |
|  | MQO_idx_ T0 | .046 | .118 | .419 |  |
|  | Sex | .030 | .038 | .750 |  |
|  | Age | .008 | .116 | .267 |  |
|  | GPAQ | < .001 | -.063 | .513 |  |
|  | MNA | .014 | .054 | .563 |  |
|  | SBP | .002 | .103 | .402 |  |
|  | DBP | -.003 | -.074 | .546 |  |
|  | HR | -.003 | -.087 | .352 |  |
|  |  |  |  |  |  |
| **F2 Muscle capacity** | Baseline F2 | -.160 | -.236 | .254 | -.007 |
|  | MQO_idx_ T0 | -.001 | -.001 | .993 |  |
|  | Sex | .377 | .291 | .164 |  |
|  | Age | .001 | .012 | .907 |  |
|  | GPAQ | < .001 | .165 | **.085** |  |
|  | MNA | .026 | .064 | .485 |  |
|  | SBP | -.005 | -.141 | .239 |  |
|  | DBP | .007 | .132 | .279 |  |
|  | HR | -.005 | -.075 | .418 |  |
|  |  |  |  |  |  |
| **F3 Functional  performance** | Baseline F3 | -.106 | -.168 | .153 | .033 |
|  | MQO_idx_ T0 | .045 | .076 | .646 |  |
|  | Sex | -.026 | -.022 | .853 |  |
|  | Age | -.015 | -.142 | .183 |  |
|  | BMI | .001 | .004 | .972 |  |
|  | GPAQ | < .001 | -.246 | **.011** |  |
|  | Nutrition Index | .053 | 1.039 | .585 |  |
|  | MNA | -.028 | -.077 | .410 |  |
|  | SBP | .001 | .048 | .693 |  |
|  | DBP | -.001 | -.025 | .839 |  |
|  | HR | -.001 | -.025 | .788 |  |
|  | PhA | -.901 | -1.009 | .597 |  |
|  |  |  |  |  |  |
| **F4 Pain & HRQoL** | Baseline F4 | -.534 | -.522 | **< .001** | .192 |
|  | MQO_idx_ T0 | .066 | .064 | .751 |  |
|  | Sex | -.048 | -.023 | .855 |  |
|  | Age | -.034 | -.178 | **.068** |  |
|  | BMI | .008 | .037 | .778 |  |
|  | GPAQ | < .001 | .044 | .610 |  |
|  | Nutrition Index | -.152 | -1.699 | .327 |  |
|  | MNA | .001 | .001 | .988 |  |
|  | SBP | .002 | .042 | .707 |  |
|  | DBP | < .001 | -.001 | .995 |  |
|  | HR | -.007 | -.069 | .414 |  |
|  | PhA | 2.567 | 1.625 | .349 |  |
|  |  |  |  |  |  |
| **MQO_idx_** | MQO_idx_ T0 | -.185 | -.323 | **.003** | .067 |
|  | Sex | .251 | .219 | **.037** |  |
|  | Age | -.013 | -.122 | .192 |  |
|  | GPAQ | < .001 | -.041 | .658 |  |
|  | MNA | -.005 | -.012 | .889 |  |
|  | SBP | -.001 | -.030 | .793 |  |
|  | DBP | .003 | .061 | .604 |  |
|  | HR | -.008 | -.151 | **.089** |  |

Values are reported as standardized regression coefficients (β), *p*-values, and adjusted *R*² for the final models. Sex was coded as 0 = female and 1 = male. Higher domain scores indicate better baseline status. BMI, Nutrition Index, and PhA were excluded from the regression models for ‘body composition’, ‘muscle capacity’ and MQO_idx_ to reduce redundancy and multicollinearity. Bold text - trend-level associations indicated by *p* < .10.

Negative adjusted R² values were observed for ‘body composition’ and ‘muscle capacity’, indicating that the models provided no explanatory value for changes in these domains.

BMI – Body Mass Index; GPAQ – Global Physical Activity Questionnaire; Nutrition Index – Nutritional Index Score; MNA – Mini Nutritional Assessment; SBP – systolic blood pressure; DBP – diastolic blood pressure; HR – heart rate; PhA – phase angle.

**Supplementary Table S7.** Baseline comparison of included participants versus not included participants

| **Baseline Parameter (T0)** | **Included (N=126) Mean ± SD** | **Not included (N=39) Mean ± SD** | **p-value** |
| --- | --- | --- | --- |
| Age (years) | 67.71 ± 5.25 | 70.76 ± 9.70 | .067 |
| Sex (Women %) | 62.7% | 56.4% | .481 |
| BMI (kg/m²) | 28.09 ± 4.52 | 26.75 ± 3.91 | .104 |
| F1: Body Fat (kg) | 27.6 ± 8.6 | 24.1 ± 8.1 | **.031** |
| F1: ECM/BCM ratio | 1.16 ± 0.24 | 1.15 ± 0.21 | .865 |
| F2: FFMI (kg/m²) | 18.11 ± 2.35 | 18.09 ± 1.95 | .949 |
| F2: BCM (kg) | 23.50 ± 4.18 | 24.22 ± 3.96 | .351 |
| F2: Handgrip Strength (kg) | 34.09 ± 9.62 | 34.94 ± 9.10 | .626 |
| F2: Quadriceps Torque (Nm) | 130.12 ± 40.17 | 126.20 ± 32.90 | .597 |
| F3: 30-second Chair Stand (reps) | 16.54 ± 4.40 | 18.06 ± 4.93 | .081 |
| F3: Timed Up and Go (s) | 6.10 ± 0.90 | 5.82 ± 1.45 | .172 |
| F3: 10-m walking speed, normal (m/s) | 1.26 ± 0.17 | 1.29 ± 0.15 | .394 |
| F3: 10-m walking speed, fast (m/s) | 1.70 ± 0.23 | 1.77 ± 0.26 | .116 |
| F4: EQ-5D Pain | 1.65 ± 0.63 (N=126) | 1.66 ± 0.67 | .954 |
| F4: EQ-5D Index | 0.937 ± 0.062 | 0.939 ± 0.062 | .908 |
| F4: SF-12 MCS | 79.62 ± 10.96 | 81.65 ± 10.21 | .333 |
| GPAQ physical activity (MET-min/wk) | 5396.73 ± 3301.13 | 5077.06 ± 3228.98 | .615 |
| MNA Score | 27.41 ± 1.53 | 27.62 ± 1.42 | .481 |
| Systolic Blood Pressure (mmHg) | 138.74 ± 18.45 | 138.22 ± 19.29 | .888 |
| Diastolic Blood Pressure (mmHg) | 81.06 ± 11.16 | 81.42 ± 11.18 | .866 |
| Resting Heart Rate (bpm) | 70.25 ± 10.23 | 72.94 ± 7.42 | .159 |
| Phase Angle (PhA, °) | 5.60 ± 0.64 | 5.62 ± 0.60 | .909 |

Values are presented as mean ± SD unless otherwise indicated. Between-group comparisons were performed using independent-samples t-tests for continuous variables and Pearson’s chi-square test for sex. Bold values indicate statistical significance at *p* < .05.
